# Supplementary material for: Sea-level records from the U.S. mid-Atlantic constrain Laurentide Ice Sheet extent during Marine Isotope Stage 3
Source: Nat Commun. 2017 May 30;8:15612. doi: 10.1038/ncomms15612 (PMC5459986; doi:10.1038/ncomms15612)
Supplement: Supplementary Information — Supplementary Notes, Supplementary Tables, Supplementary Figures and Supplementary References [file ncomms15612-s1.pdf]

Supplementary Note 1: Sea-level data

In Supplementary Table 1 we list the elevation data from the U.S. mid-Atlantic coast used in Figure 1 to derive constraints on peak sea level during MIS 5a (80 ka) and mid-MIS 3 (50-35 ka). Parham et al.[1] compiled previously published data and assigned depositional environments to observed facies in cores and outcrops. In Figure 1, and Supplementary Table 1, markers are labeled as terrestrial, marine, or tidal. All dates are based on OSL except those noted as U-series, AAR (amino-acid racemization), or C^{14}. Marine limiting samples GH25, GH15, MYK01 are dated to 37.7 ± 3.6 ka, 40 ± 4.3 ka, and 45.8 ± 4.3 ka, respectively; these records constrain RSL from 50-35 ka to being above -0.9 m, -3 m, and -2 m, respectively, as these correspond to shallow marine facies. GH 15/25 is a shelly muddy sand and has mollusk shell fragments, whereas MYK01 is a shelly sand in which *Melita* has been observed (interpreted as a shallow marine/shoreline complex). Qtp-1 and TCK-19 are terrestrial limiting samples, both at 0.8 m elevation dated to 39.6 ± 6.6 ka and 45.3± 4.3 ka, respectively, and correspond to quartz lithofacies, identified by Parham et al.[1] as terrestrial (see Scott et al.[2]). These two samples represent the minimum elevation of terrestrial sea-level indicators whose age uncertainties span 50-35 ka. We therefore conclude that RSL was in the range -1 to 1 m from 50-35 ka (as in Figure 1).

The data of MIS 5a age from the mid-Atlantic coast of the U.S. suggest that local relative sea level (RSL) was between +2.55 and +7.5 m. Ages determined by Parham et al.[1] include samples FNB-02 and GH10, which are dated to 84.4 ± 8.6 ka and 81.5 ± 6.9 ka, at elevations of 2.55 m and 7.5 m respectively. FNB-02 is a marine limiting indicator, corresponding to a shelly muddy sand, interpreted as shallow marine. GH10-01 is a terrestrial-limiting indicator in a sand facies, interpreted as “shoreline complex”[1]. While other sea level markers are lower than this range during this time interval, given the associated large age uncertainties, we assume the higher range represents the MIS 5a highstand. Moreover, this sea-level highstand is in agreement with previous studies of MIS 5a RSL in the region[3].

We add an uncertainty of ± 3 m to the elevation of these sea-level indicators since the tidal range may have been different in the past relative to the Holocene. For instance, Hill et al.[4] (2011) showed that the tidal range of the mid-Atlantic U.S. coast might have been 2-3 times the magnitude at 9 ka. Present day tidal range in the region is ~1 m[5], and we thus assign a maximum value of ± 3 m to account for the possible paleo tide range.

Supplementary Table 1: Sea-level data

Sample No.	Elevation (m)	Age (ka)	Age error (ka)	Indicator type	Analytical technique	Latitude	Longitude	Reference
FS181	9.3	35.8	6.1	terrestrial	OSL	35.609	-77.217	6
KNL02	5.5	41.8	3.2	terrestrial	OSL	35.022	-76.898	1
GH23-01	3.65	31.8	3	terrestrial	OSL	36.717	-76.529	1
GH25-01	-0.9	37.7	3.6	marine	OSL	36.699	-76.359	1
GH15-01	-3	40	4.3	marine	OSL	35.737	-76.534	1
MYK-01	-2	45.8	4.3	marine	OSL	36.532	-76.177	1
TCK-17	0.345	54.3	6.7	terrestrial	OSL	36.270	-75.911	7
TCK-19	0.826	45.3	4.3	terrestrial	OSL	36.335	-75.907	7
HS-02	-0.15	46.4	2.5	terrestrial	OSL	36.343	-76.064	7
HS-03	-0.9	48.9	10.9	terrestrial	OSL	36.343	-76.064	7
RI-03	0.05	55.4	7.2	terrestrial	OSL	35.939	-75.718	7
DR-2	1.5	62	12	tidal	OSL	35.930	-76.165	1
qw-1	1.9	46.9	6.9	terrestrial	OSL	37.162	-75.980	8
qw-2	1.9	38.9	5.5	terrestrial	OSL	37.162	-75.980	8
qts-1	5.1	49.7	5.9	terrestrial	OSL	36.895	-76.515	8
qtp-1	0.8	39.6	6.6	terrestrial	OSL	36.577	-76.037	8
qtp-2	0.75	44.4	5.2	terrestrial	OSL	36.577	-76.037	8
cr-08-gp1b	3.6	50.4	5.8	terrestrial	OSL	34.729	-76.861	9
c-1	-2.8	62	2	marine	U-series	36.975	-76.172	10
ELP-05	-0.6	66.9	6.7	tidal	OSL	35.857	-75.952	1
SS-2 exp	2.85	79.81	4.55	terrestrial	OSL	34.977	−75.830	7
FNB-02	2.55	84.4	8.6	marine	OSL	34.982	-76.945	1
CR-08-GP3	4	80.8	9	terrestrial	OSL	34.729	-76.861	9
CR-08-GP5	4.5	83.4	8.92	terrestrial	OSL	34.729	-76.861	9
GH18-01	-3.4	76.9	7.1	terrestrial	OSL	35.620	-76.357	1
GH2-01	11.5	88.5	8	terrestrial	OSL	35.167	-76.851	1
MLD01-02a	-7	74.9	8	marine	OSL	35.509	-76.001	1
MLD01-02b	-8.5	80	10	tidal	AAR	35.509	-76.001	1
GH8-02	12.2	80.2	7	terrestrial	OSL	35.732	-76.815	1
GH10-01	7.5	81.5	6.9	terrestrial	OSL	35.715	-76.757	1
TT-01	12.81	87.5	6.9	terrestrial	OSL	36.346	-76.602	1
qnb-1	3.25	69.3	7.6	terrestrial	OSL	37.195	-76.005	8
qnb-2	3.25	69.7	8.4	terrestrial	OSL	37.195	-76.005	8
GH26,30, 33	5.8	80	10	marine	AAR	35.726	-76.099	1
C-2	-1	73	4	marine	U-series	36.742	-76.188	10
4	0	75	5	marine	U-series	36.788	-76.196	11
GH20-01	4.7	94.1	8.3	terrestrial	OSL	36.392	-76.530	1

CR-08-GP4	7.16	95.2	9.25	terrestrial	OSL	34.729	-76.861	9
GH12-01	5.3	106.7	9.3	tidal	OSL	35.703	-76.755	1
GH24-01	2.04	97.4	8.6	marine	OSL	36.729	-76.453	1
GH5-01	-0.05	102.3	9.9	marine	OSL	35.179	-76.771	1
EDP-03	10	103	8.3	tidal	OSL	35.250	-76.771	1
ELP-01	-7.3	100.3	9.5	marine	OSL	35.900	-75.963	1
GH1-01	7.42	111.6	10.2	tidal	OSL	35.182	-76.857	1
BC-02	11.1	130	10.8	tidal	OSL	35.756	-76.830	1
OBX-12	-16.25	43.2	0.48	marine	14 C	35.439	-75.486	12

Supplementary Note 2: Decomposing Sea Level Change into Deformational and Direct Gravitational Effects

Relative sea level (*RSL*) can be decomposed into global mean sea level (*GMSL*) plus a term associated with glacial isostatic adjustment (*GIA*):

$$RSL = GMSL + GIA, \quad (1)$$

and the *GIA* term can be further decomposed into sea level contributions from crustal deformation (including the gravitational perturbation associated with this deformation; *R*) and the direct gravitational effects of the surface mass load (G_D):

$$GIA = R + G_D \quad (2)$$

Figure S1 shows the decomposition of RSL predictions at our reference site in the Albemarle Embayment region (white star, Figure 1) into the three terms *GMSL*, *R* and G_D. Results are shown for simulations based on ice histories ICE_{PC} and ICE_{PC2}. Delaying the growth of ice in the eastern sector of the Laurentide Ice Sheet (LIS) until MIS 3 (44 ka), suppresses deformational effects, yielding a higher RSL prediction for the ICE_{PC2} model at 44 ka relative to the ICE_{PC} simulation.

The relative contribution of crustal deformation to predicted RSL will be a function of the Earth model, and in particular the values adopted for lithospheric thickness and lower and upper mantle viscosities. Using the Earth model described in the main text, we noted that crustal deformation dominates the RSL signal compared to direct gravitational effects. We explore the sensitivity of this decomposition to variations in Earth structure by running simulations for additional Earth models. In Supplementary Figure 2, results are shown for predictions in which the following parameter ranges are considered: (A) lithospheric thickness from 72 - 127 km; (B) lower mantle viscosity from [5-30] x 10^{21} Pa s; and (C) upper mantle viscosity from [0.3-1] x 10^{21} Pa s. We note that several of these Earth models predict local RSL values that lie outside the observational bound at MIS 3 (Supplementary Figure 2, orange box). For predictions that are consistent with this bound, the crustal deformation signal is larger than the signal from direct gravitational effects of the surface mass load

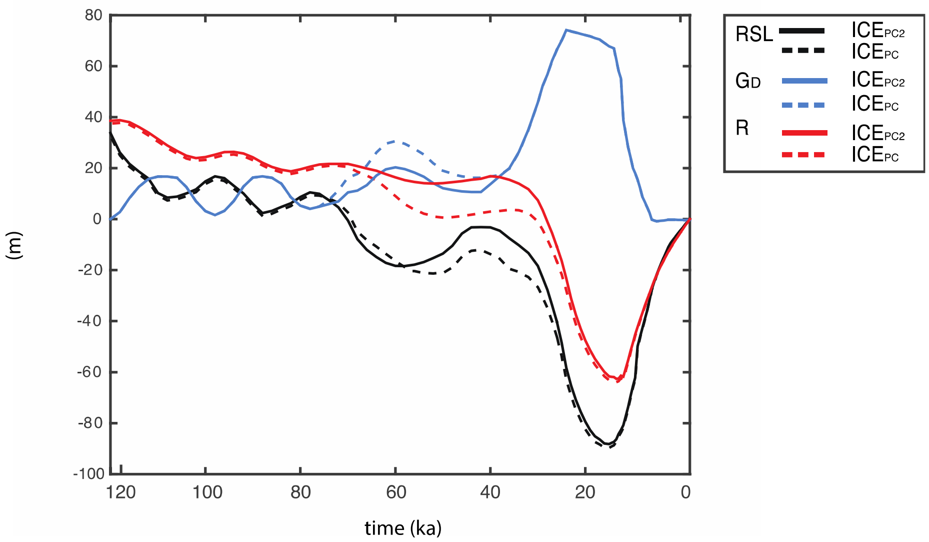

Supplementary Figure 1 | Decomposition of RSL predictions (black lines) at location shown by white star (Figure 1) is based on the ice histories ICE_{PC2} (solid lines) and ICE_{PC} (dashed lines) into contributions from deformation (R; red lines) and direct gravitational attraction of the surface mass load (G_D; blue lines).

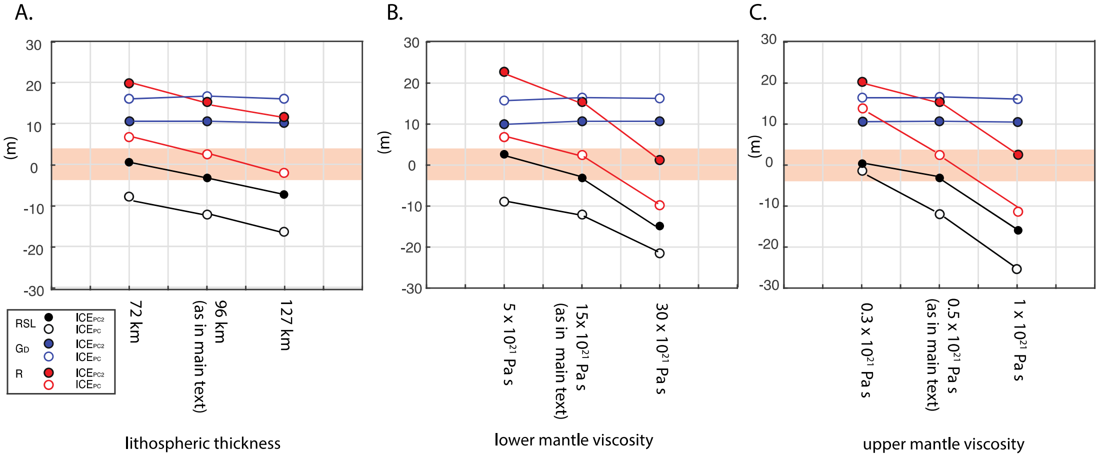

Supplementary Figure 2| Decomposition of RSL predictions (black) at location shown by white star (Figure 1) is based on the ice histories ICE_{PC2} (solid circles) and ICE_{PC} (white circles) into contributions from deformation (R; red) and direct gravitational attraction of the surface mass load (G_D; blue) for six additional Earth models where we perturb A. the lithospheric thickness B. lower mantle viscosity C. upper mantle viscosity. The orange box denotes the observational sea-level bounds (Figure 1).

Supplementary Note 3: Sensitivity to ice history and geographic distribution

To test the sensitivity of our simulations to the GMSL values at MIS 5a and MIS 5c, we constructed ice histories as described above, with the exception that we allowed GMSL to range from -16 to 0 m at 80 ka (MIS 5a), and from -20 to 0 m at 100 ka (MIS 5c). We ran simulations with 100 of these ice models and found that the predicted RSL at 44 ka is perturbed by no more than ~0.7 m.

Next we explored the sensitivity of sea-level predictions in the Albemarle Embayment region to variations in the ice cover over the eastern sector of the LIS (Figure S3). Specifically, we: (1) shifted the northern latitudinal boundary of the no-ice zone in eastern Laurentia from 60 °N (ICE_{PC2}) to 57°N (ICE_{PC3}) and 55°N (ICE_{PC4}); and (2) modified the location of ice in eastern Laurentia, such that Newfoundland and Northern Quebec are glaciated (Geometry 1,2, and 3). For this series of ice geometries, we include one ice history where Baffin Island is glaciated (Geometry 1), and two where the southern portion of the island is not (Geometries 2 and 3). The latter two ice geometries are distinguished by their latitudinal bounds: in Geometry 2, ice cover extends to 51°N in the eastern sector of the LIS, whereas in Geometry 3 it extends to 52°N. Supplementary Table 2 gives the change in ice volume compared with the standard assumption of ice distribution for each ice model listed, and the resulting relative sea level predicted.

Supplementary Table 2: Prediction of relative sea level at 44 ka based on a variety of ice models

Ice model	Difference in ice volume (m GMSL)	Relative Sea Level
ICE_{PC}	0	-12
ICE_{PC2}	6.8	-3.1
ICE_{PC3}	5.3	-3.8
ICE_{PC4}	4.2	-5.1
Geometry 1	5.3	-6.3
Geometry 2	6.1	-5.8
Geometry 3	6.53	-3.8

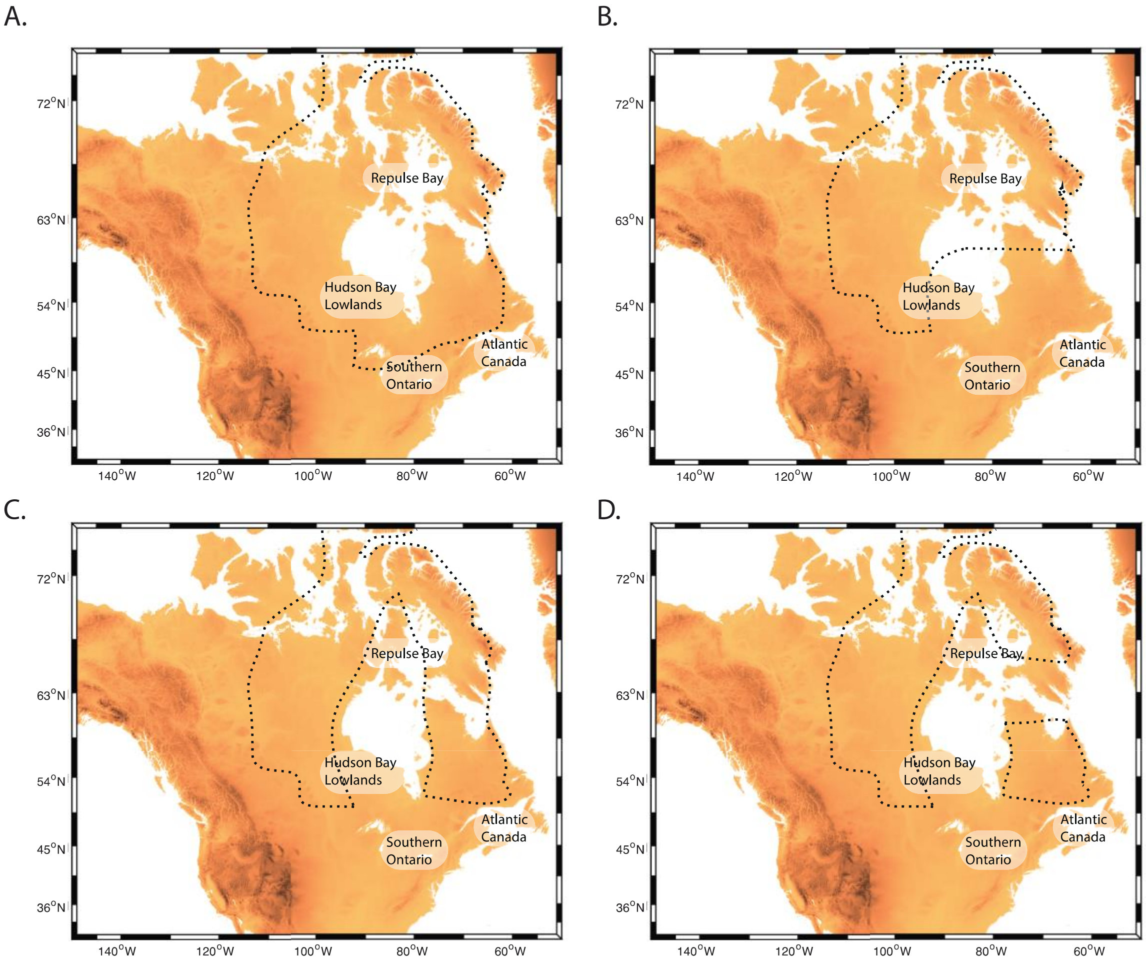

Supplementary Figure 3 | Geographic distribution of ice cover for various ice models. A. ICE$_{PC}$ (standard ice distribution, as in Figure 2 and 3B) B. ICE$_{PC2}$ (alternate ice distribution, as in Figure 2 and 3B). ICE$_{PC3}$ and ICE$_{PC4}$ are distinguished by the latitudinal extent of ice cover over the eastern sector of the LIS (see text). C. Geometry 1. D. Geometry 2 & 3 (these two models are distinguished by the latitudinal extent of ice cover - see text). In frames B-D, the difference in ice volume between the given ice model and model ICE$_{PC}$ in eastern Laurentia is distributed uniformly over the LGM extent of western Laurentia, the Cordilleran Ice Sheet, and Fennoscandia.

Supplementary Note 4: Earth model sensitivity

We ran a large suite of simulations in which we adopted ice histories with the GMSL curves shown in Figure 3A. The calculations, shown in Figure 3B, were based on an Earth model with upper and lower mantle viscosities of 0.5 x 10^{21} Pa s and 15 x 10^{21} Pa s, respectively. These runs yielded predicted peak RSL values within the Albemarle Embayment region during MIS 3 that ranged from -28.6 to -5.7 m when pre- and post-LGM ice geometries were assumed to match when GMSL values were equal (as in ice model ICE_{PC}) and -14.8 m to 2.4 m when it was assumed that the eastern sector of the LIS remained ice free in an extended period leading to MIS 3 (as in ice model ICE_{PC2}). When we reran these calculations using an Earth model in which the lower mantle viscosity was increased to 2 x 10^{22} Pa s, the above ranges were perturbed upwards by ~1.5 m. Decreasing the lower mantle viscosity to 10^{22} Pa s, perturbed the above RSL range downwards by ~3 m (Supplementary Figure 3).

To explore the sensitivity of our results to other Earth model parameters, we ran simulations using a suite of Earth models where we varied values of the lithospheric thickness and upper and lower mantle viscosity. The resulting RSL predictions for MIS 3, based on the ice model ICE_{PC2} (Figure 2A), for the representative site in Albemarle Embayment (white star, Figure 1) are plotted in Figure S5. In the figure, results are shown for predictions in which the following Earth model parameters were adopted: (A) lithospheric thickness of 72 km (dark blue), 96 km (white) and 127 km (pink); (B) upper mantle viscosity of 0.3x10^{21} km (dark blue), 0.5x10^{21} Pa s (white), and 1x10^{21} Pa s (pink); and (C) lower mantle viscosity of 5x10^{21} km (dark blue), 15x10^{21} Pa s (white) and 30x10^{21} Pa s (pink).

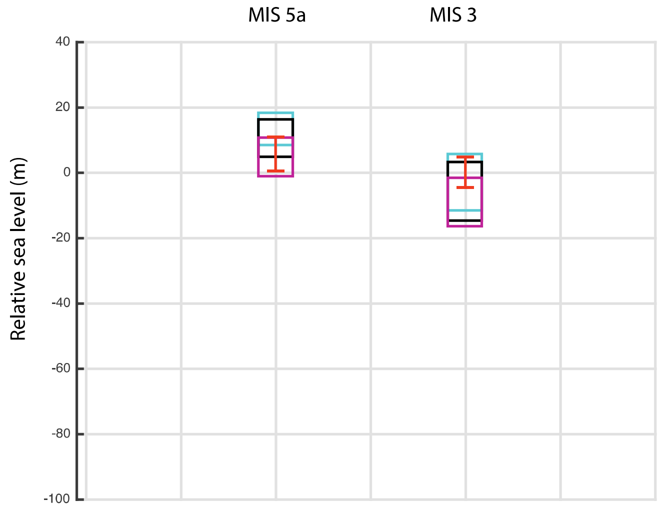

Supplementary Figure 4 | Range of RSL highstand predictions during MIS 5a and MIS 3 based on simulations with the GMSL scenarios in Figure 3A and a lower mantle viscosity of either 10^{22} Pa s (pink rectangle) or 2 x 10^{22} Pa s (blue rectangle). The black rectangles indicate the range of RSL values predicted in main text (lower mantle viscosity of 1.5 x 10^{22} Pa s; Figure 3B). The orange bars at 80 ka and 44 ka show the observational constraints (Figure 1). All calculations are based on ice models that assume that the eastern sector of the LIS remained ice free in the period 80-44 ka (similar to the ICE_{PC2} model).

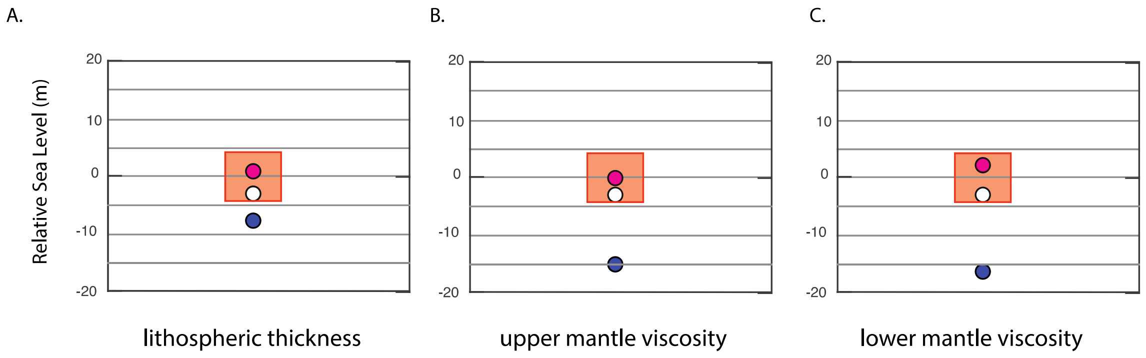

Supplementary Figure 5 | Relative sea level predictions at location shown by white star (Figure 1) are based on the ice history ICE_{PC2} (Figure 2A) with seven different Earth models (including that adopted in main text). A. Lithospheric thickness is increased to 127 km (pink) and decreased to 72 km (dark blue). B. Upper mantle viscosity is increased to 1 $x10^{21}$ Pa s (pink) and decreased to $0.3x10^{21}$ Pa s (dark blue). C. Lower mantle viscosity is increased to 30 $x10^{21}$ Pa s (pink) and decreased to 5 $x10^{21}$ Pa s (dark blue). The Earth model adopted in the main text is shown by the white circle, and the observational constraints on the MIS 3 sea-level highstand is shown by the orange box (Figure 1).

Supplementary References

1. Parham, P. R. *et al.* Quaternary coastal lithofacies, sequence development and stratigraphy in a passive margin setting, North Carolina and Virginia, USA. *Sedimentology* **60,** 503–547 (2013).
2. Scott, T. W., Swift, D. J. P., Whittecar, G. R. & Brook, G. A. Glacioisostatic influences on Virginia's late Pleistocene coastal plain deposits. *Geomorphology* **116,** 175–188 (2010).
3. Wehmiller, J. F., Edwards, R. L. & Martin-mcnaughton, J. Uranium-series coral ages from the US Atlantic Coastal Plain – the '" 80 ka problem "' revisited. (2004).
4. Hill, D. F., Griffiths, S. D., Peltier, W. R., Horton, B. P. & T??rnqvist, T. E. High-resolution numerical modeling of tides in the western Atlantic, Gulf of Mexico, and Caribbean Sea during the Holocene. *J. Geophys. Res. Ocean.* **116,** 1–16 (2011).
5. Titus, J., & Wang, J. Maps of Lands Close to Sea Level along the Middle Atlantic Coast of the United States. United States Environmental Protection Agency. *2008*
6. Moore, C. Geoarchaeological Investigations of Stratified Holocene Aeolian Deposits along the Tar River in North Carolina. (Coastal Resources Management, East Carolina University, Greenville, NC, 307 pp. Murray, 2009).
7. Mallinson, D., Burdette, K., Mahan, S. & Brook, G. Optically stimulated luminescence age controls on late Pleistocene and Holocene coastal lithosomes, North Carolina, USA. *Quat. Res.* **69,** 97–109 (2008).
8. Scott, T. W. (2006). Correlating late Pleistocene Deposits on the Coastal Plain of Virginia with the Glacial-Eustatic Sea-Level. (Old Dominion University, Norfolk, VA, 111, 2006).
9. Best, K. M. (2010). Quaternary geologic evolution of the Croatan beach ridge complex, Bogue Sound, and Bogue Banks, Carteret County, NC. (Department of Geological Sciences, East Carolina University, Greenville, NC, 265 pp. Blackwelder, 2010).
10. Mixon, R. ., Szabo, B. J. & Owens, J. P. Uranium-Series Dating of Molltisks aad Corals , arid Age of Pleistocene Deposits ^ Chesapeake Bay Area , Virginia and Maryland Uranium-Series Dating of Mollusks and Corals , and Age of Pleistocene Deposits , Chesapeake Bay Area , Virginia and Maryland.
11. Cronin, T. M., Szabo, B. J., Ager, T. A., Hazel, J. E. & Owens, J. P. Quaternary climates and sea levels of the u.s. Atlantic coastal plain. *Science* **211,** 233–40 (1981).
12. Culver, S. J. *et al.* Micropaleontologic record of Quaternary paleoenvironments in the Central Albemarle Embayment, North Carolina, U.S.A. *Palaeogeogr. Palaeoclimatol. Palaeoecol.* **305,** 227–249 (2011).
